# *SLA1* governs azole resistance in *Candidozyma auris* via mitochondrial ROS-driven efflux pump expression

**DOI:** 10.3389/fmicb.2026.1948008

**Published:** 2026-10-07

**Authors:** Chenhao Suo, Wanli Zhang, Yuhan Li, Chao Li, Yaning Cui, Yuwei Xiao, Jianjun Lei, Yiru Gao, He Zhang

**Affiliations:** 1 Department of Laboratory Animal Center, General Hospital of Northern Theater Command, Shenyang, Liaoning, China; 2 The State Key Laboratory of Pharmaceutical Biotechnology, Chemistry and Biomedicine Innovation Center (ChemBIC), Division of Immunology, Medical School, Nanjing University, Nanjing, Jiangsu, China; 3 College of Animal Science and Veterinary Medicine, Shenyang Agricultural University, Shenyang, China; 4 Department of Emergency Medicine, The Second Affiliated Hospital of Army Medical University, Chongqing, China; 5 State Key Laboratory for Diagnosis and Treatment of Infectious Diseases, The First Hospital of China Medical University, Shenyang, China; 6 College of Life and Health Science, Northeastern University, Shenyang, Liaoning, China

**Keywords:** *Candidozyma auris*, *CDR1*, drug-resistant, reactive oxygen species, *SLA1*, virulence

## Abstract

**Introduction:**

*Candidozyma auris* is an emerging multidrug-resistant fungal pathogen, and widespread fluconazole (FLC) resistance severely limits clinical treatment options. Overexpression of the efflux transporter *CDR1* represents a major driver of azole resistance; however, how oxidative stress modulates this process remains poorly understood. This study aimed to identify novel regulators that connect oxidative stress adaptation with azole resistance in *C. auris*.

**Methods:**

We constructed *SLA1* deletion mutants of *C. auris*. *SLA1* encodes a conserved regulator of cytoskeletal organization and membrane trafficking. We evaluated mitochondrial homeostasis, mtROS accumulation, and *CDR1* expression. Pharmacological mitochondrial perturbation was used to disentangle the individual effects of ROS accumulation and mitochondrial dysfunction on FLC resistance. We further assessed cell envelope integrity, *in vivo* virulence, and the transcriptional correlation between *SLA1* and *CDR1* using clinical isolates.

**Results:**

*SLA1* deletion triggered mitochondrial dysfunction, increased mtROS accumulation, upregulated *CDR1*, and enhanced azole resistance. Pharmacological disruption of mitochondria further raised ROS levels yet suppressed *CDR1*, depleted ATP, accumulated NADH, and abolished FLC resistance. This indicates that ROS elevation alone cannot sustain resistance under severe mitochondrial impairment. *SLA1* knockout compromised cell envelope integrity and attenuated in vivo virulence, revealing a fitness trade-off during antifungal adaptation. Clinical isolates exhibited a significant inverse correlation between *SLA1* and *CDR1* transcript levels.

**Discussion:**

This study identifies SLA1 as a previously uncharacterized modulator of oxidative stress adaptation and azole susceptibility in *C. auris*. Mitochondrial functional status governs the relationship between oxidative stress and azole resistance. The *SLA1*-dependent axis promotes antifungal resistance at the expense of reduced virulence. These findings reveal a new mechanism of drug adaptation in *C. auris* and point to a potential target for combating multidrug-resistant *C. auris*.

## Introduction

*Candidozyma auris* (*C. auris*) has emerged over the past decade as a major global health threat, characterized by rapid transmission in healthcare settings, high mortality rates, and a pronounced capacity for multidrug resistance (Eix and Nett, 2025; Suphavilai et al., 2024; Wasylyshyn and Stoneman, 2024). Among antifungal agents, fluconazole (FLC) remains one of the most widely used first-line therapies; however, resistance to FLC is highly prevalent in clinical isolates, frequently exceeding 80–90% (Eix and Nett, 2025; da Silva et al., 2025). Although previously established mechanisms, such as mutations in *ERG11* and the overexpression of efflux transporters, including *CDR1*, contribute to azole resistance, these genetic determinants alone do not fully explain the remarkable adaptability and variability of resistance observed in *C. auris* (Barker et al., 2025a; Chow et al., 2023; Lass-Flörl et al., 2024). This discrepancy suggests that non-genetic and environment-responsive mechanisms play a critical role in shaping antifungal resistance.

During infection, fungal pathogens encounter diverse host-derived stresses, among which oxidative stress represents a central selective pressure (Allert et al., 2022; Phan-Canh et al., 2026; Wani et al., 2024). Reactive oxygen species (ROS), generated as a part of the host immune response, have traditionally been viewed as cytotoxic molecules that damage cellular components (Dong et al., 2022; Liu et al., 2022; Liu et al., 2025). However, accumulating evidence indicates that ROS functions as a signaling intermediate that regulate stress adaptation, metabolism, and drug tolerance in fungi (Liu et al., 2025). Notably, ROS are produced by multiple intracellular sources, including NADPH oxidases, peroxisomes, and the endoplasmic reticulum, in addition to the mitochondria (Cheung and Vousden, 2022; Zhang et al., 2025b). This multiplicity raises an important unresolved question about whether ROS universally promotes antifungal resistance or whether their functional consequences depend on the cellular context in which they are generated (Czajka et al., 2023; Wang et al., 2024; Zhang et al., 2025a).

Among these sources, the mitochondria occupy a unique position by coupling ROS production to cellular metabolic and energetic states (Ahmed Selim and Wojtovich, 2025; DiGiovanni et al., 2025; Du et al., 2024). In addition to serving as a major site of intracellular ROS generation, the mitochondria are central to ATP production, redox balance, and biosynthetic processes that underpin essential cellular functions (Delaunay et al., 2022; Du et al., 2025; Quintana-Cabrera and Scorrano, 2023; Wai, 2024). In this context, it remains unclear whether mitochondrial ROS acts as a sufficient driver of resistance signaling or whether the ability of fungal cells to execute resistance programs depends on the integrity of mitochondrial function (Phan-Canh et al., 2026; Li et al., 2024; Song Y. et al., 2026). Addressing this question requires identifying regulatory factors that connect stress responses to cellular organization and mitochondrial homeostasis (Obara et al., 2024; Prashar et al., 2024; Sohn et al., 2023). Proteins involved in cytoskeletal dynamics and membrane trafficking are particularly well positioned to fulfill such roles, as they coordinate intracellular architecture and organelle function (Nie et al., 2024; Song R. et al., 2026). In this context, transcriptomic analysis of oxidative stress responses in *C. auris* revealed that *SLA1* (*B9J08_02104*), a conserved regulator of actin organization and endocytic trafficking, is significantly downregulated under oxidative conditions. Considering its established roles in maintaining cellular structure and membrane dynamics, this observation suggests that *SLA1* may serve as a nexus connecting stress-induced cellular remodeling with mitochondrial function.

Based on these observations, we hypothesized that *SLA1* contributes to cellular adaptation during oxidative stress and influences fluconazole susceptibility. We therefore investigated how *SLA1* deletion alters mitochondrial physiology, oxidative stress responses, resistance-associated gene expression and antifungal susceptibility, with the aim of characterizing the relationship among these processes rather than defining a direct signaling pathway. This approach enables us to move beyond a linear ROS-centric model and instead interrogate how cellular context determines the functional consequences of oxidative stress in *C. auris.*

## Results

### Oxidative stress drives FLC resistance and identifies *SLA1* as a key candidate

Oxidative stress is a key host-derived cue regulating antifungal susceptibility in invasive fungi, albeit its role in FLC resistance in *C. auris* remains poorly defined (Blancett et al., 2021; Yang et al., 2025). To address this gap, spotting assays revealed that FLC resistance of *C. auris* was reduced by the ROS scavenger N-acetylcysteine (NAC) and enhanced by H₂O₂-induced oxidative stress (Figure 1A). This phenotype was fully validated via growth curve analysis (Figure 1B).

**Figure 1:**
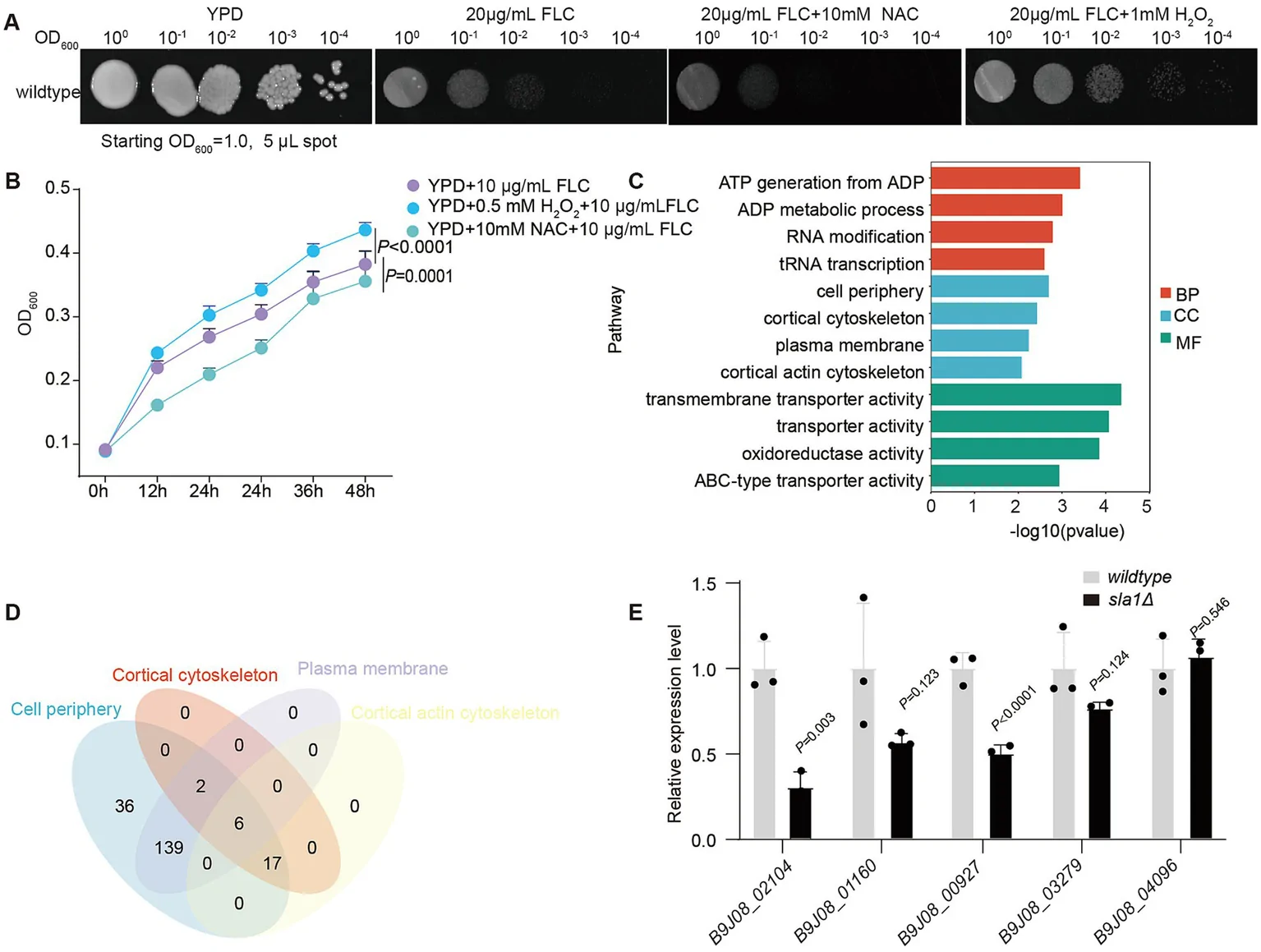
Oxidative stress regulates FLC resistance and identifies SLA1 as a key candidate in *C. auris.* **(A)** Spotting assay of *C. auris* strain. Cells were grown overnight in YPD medium at 30 °C, Strains were adjusted to OD₆₀₀ = 1.0 (~1 × 10^7^ cells/mL). We performed 5-fold serial dilutions (10^0^, 10^−1^, 10^−2^, 10^−3^, 10^−4^), and 5 μL of each dilution was spotted onto YPD agar supplemented with the FLC, NAC + FLC, and H₂O₂ + FLC and the plates were incubated at 30 for 2 days. **(B)** Survival curves of *C. auris* under the conditions described in **(A)**. **(C)** GO analyses. Samples of RNA were isolated from *C. auris* cells (*n* = 3) supplemented with or without 1 mM H₂O₂. GO analysis was performed. **(D)** Venn diagram of overlapping genes from four enriched cell periphery-related terms. **(E)** RT-qPCR verification of candidate genes; The WT (*n* = 3) were incubated at YPD and YPD supplemented with the H₂O₂. Then relative expressions were determined using RT-qPCR. *ACT1* was used as the control. Two-tailed unpaired *t*-tests were used. Data are expressed as the mean ± SD.

To identify molecular mediators linking oxidative stress to FLC resistance, transcriptomic profiling was performed on *C. auris* cultured under standard yeast YPD conditions or H₂O₂ treatment. Gene ontology (GO) cellular component enrichment analysis revealed that differentially expressed genes were significantly enriched in terms associated with the cell periphery, cortical cytoskeleton, and plasma membrane, indicating oxidative stress–induced remodeling of cell surface architecture relevant to azole drug uptake and efflux (Figure 1C). Venn analysis of these enriched categories identified six overlapping candidate genes. RT-qPCR confirmed that *SLA1*, a gene with uncharacterized roles in *C. auris* stress adaptation and drug resistance, was most significantly downregulated under oxidative stress and thus selected for functional characterization (Figures 1D,E).

### Deletion of *SLA1* confers FLC resistance by inducing *CDR1* expression

Building on our observation that *SLA1* is the most significantly downregulated gene under oxidative stress and considering its established role in cortical cytoskeleton organization and membrane homeostasis in model fungi, we next generated an *SLA1*-knockout strain (*sla1Δ*) using CRISPR-Cas9–mediated genome editing to define its function in antifungal drug resistance in *C. auris*. Spot dilution assays revealed a divergent drug-susceptibility profile: *sla1Δ* showed markedly increased susceptibility to polyene amphotericin B, but exhibited robust resistance to azoles, including FLC and ketoconazole, with the strongest resistance observed for FLC (Figure 2A).

**Figure 2:**
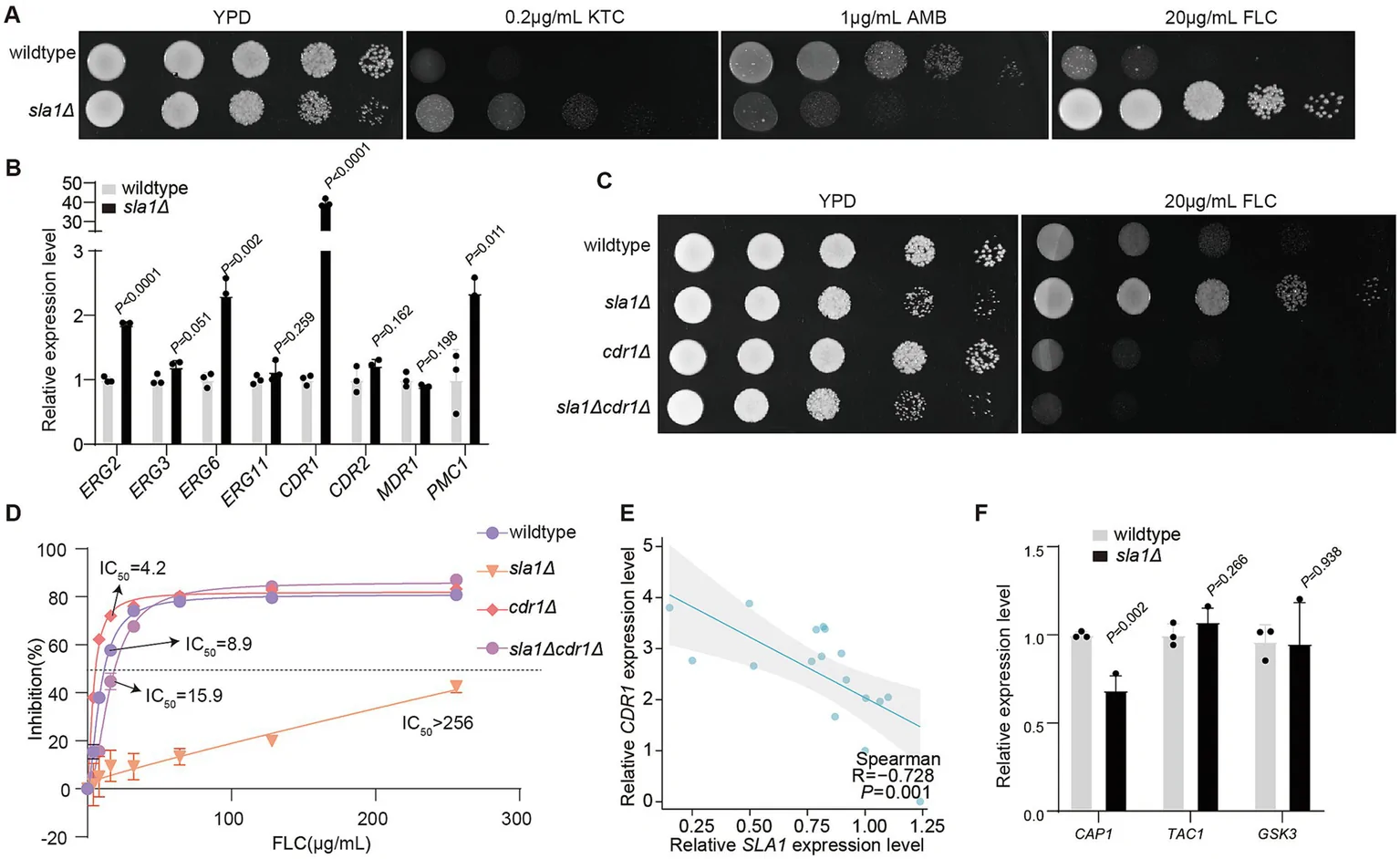
*SLA1* deletion drives FLC resistance through upregulation of *CDR1.* **(A)** Spotting assays of WT and *sla1Δ* mutants. Strains were spotted onto YPD agar supplemented with the FLC, KTC, AMB and the plates were incubated at 30 for 2 days. **(B)** RT-qPCR analysis of *ERG* genes, *CDR1*, *CDR2*, *MDR1* and *PMC1* in WT and *sla1Δ* strains (*n* = 3). **(C)** Spotting assays of WT, *sla1Δ*, *cdr1Δ*, and *sla1Δcdr1Δ* strains. Indicated strains were spotted with or without FLC. **(D)** FLC IC₅₀ values of WT, *sla1Δ*, *cdr1Δ*, and *sla1Δcdr1Δ* strains. **(E)** Correlation analysis between *SLA1* and *CDR1* transcript levels in 17 clinical *C. auris* isolates. **(F)** RT-qPCR analysis of *CDR1* regulators *CAP1*, *TAC1*, and *GSK3* in WT and *sla1Δ* strains (*n* = 3). Two-tailed unpaired *t*-tests were used for statistical analyses; data are expressed as the mean ± SD.

To investigate the molecular mechanism driving this FLC-specific resistance, we quantified the expression of core genes governing azole susceptibility, including ERG family ergosterol biosynthesis genes, the canonical azole efflux pump *CDR1*, and the vacuolar drug-sequestration pump *PMC1*. Most genes were significantly upregulated in *sla1Δ*, with *CDR1* exhibiting the highest induction (~15-fold), making it the primary candidate mediator of *SLA1*-dependent FLC resistance (Figure 2B). To validate this role, we generated a *sla1Δcdr1Δ* double-knockout strain. Spotting assays revealed that *CDR1* deletion abrogated the FLC-resistance phenotype of *sla1Δ*, confirming that *CDR1* is nonredundant and essential for *SLA1*-mediated azole resistance (Figure 2C). Quantification of FLC half-maximal inhibitory concentration (IC₅₀) values further supported this conclusion, with susceptibility ranked as follows: *cdr1Δ >* wild-type (WT) > *sla1Δcdr1Δ* > *sla1Δ*. Although *CDR1* deletion fully abrogated the prominent FLC resistance of *sla1Δ*, the *sla1Δcdr1Δ* double mutant continued to display a slightly higher FLC IC₅₀ than the wild type. This residual difference may be partially attributed to growth defects induced by dual-gene deletion (Figure 2D). To evaluate the broad clinical relevance of *SLA1*-mediated *CDR1* regulation, a random selection of 17 clinical isolates of *C. auris* was subjected to testing. Our data revealed a significant negative correlation between *SLA1* and *CDR1* transcript levels across all clinical strains(Figure 2E), Of note, further integration of MIC susceptibility data is warranted to directly link this expression pattern to clinical azole resistance phenotypes.

Finally, to determine whether *SLA1* regulates *CDR1* via known pathways, we assessed the transcript levels of established *CDR1* transcriptional regulators (*CAP1*, *TAC1*, and *GSK3*) in *sla1Δ* (Barker et al., 2025a,b; Chow et al., 2023). In *sla1Δ* cells, *CAP1* expression was downregulated, which conflicts with its canonical role as a positive regulator of *CDR1*. No changes in *TAC1* and *GSK3* expression were observed, indicating that *SLA1* modulates *CDR1* through uncharacterized alternative regulatory pathways in *C. auris* (Figure 2F).

### *SLA1* deletion disrupts mitochondrial bioenergetics and redox homeostasis to govern FLC resistance

Our earlier work established that *SLA1* deletion drives FLC resistance via *CDR1* upregulation through an alternative regulatory mechanism. Considering the established links among mitochondrial dysfunction, redox imbalance, and azole resistance in pathogenic fungi (da Silva et al., 2025), we performed transcriptomic profiling to define molecular pathways dysregulated in *sla1Δ* cells. GO enrichment analysis revealed that differentially expressed genes in *sla1Δ* were significantly enriched in functional terms associated with transmembrane transporter activity, oxidoreductase activity, metal ion transport, and cellular iron homeostasis (Figure 3A). Gene set enrichment analysis revealed significant negative enrichment of oxidoreductase activity (Figure 3B) and positive enrichment of the oxidative phosphorylation pathway (Figure 3C), linking *SLA1* loss to impaired redox homeostasis and mitochondrial respiratory dysfunction. Furthermore, modulation of cellular redox status profoundly affected FLC susceptibility: the enhanced FLC resistance observed in the sla1Δ mutant was partially reversed by co-treatment with the ROS scavenger NAC, whereas exogenous H₂O₂-induced oxidative stress further augmented this resistance (Figure 3D).

**Figure 3:**
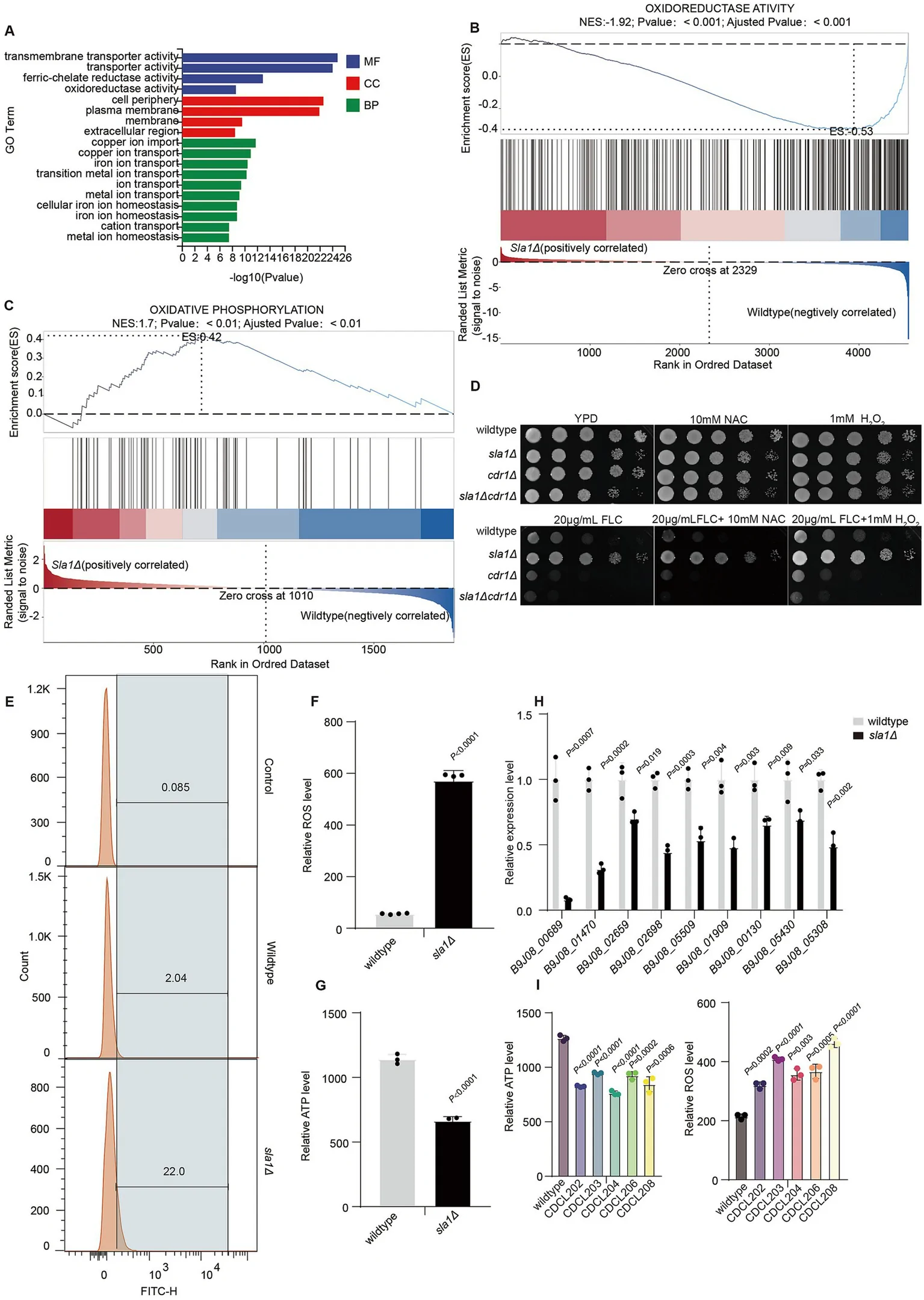
*SLA1* deletion disrupts redox homeostasis and mitochondrial energy metabolism to regulate FLC resistance in *C. auris*. **(A)** GO analyses. GO term enrichment analyses were performed using the RNA-seq data from WT and *sla1Δ* strains. **(B,C)** Gene Set Enrichment Analysis (GSEA). Comparing the transcriptome data from the WT and *sla1Δ* strains. **(D)** Spotting assays of WT and *sla1Δ* strains. Strains were spotted onto YPD agar supplemented FLC, FLC + NAC, or FLC + H₂O₂ conditions. **(E)** Flow cytometry analysis of intracellular ROS levels in WT, and *sla1Δ* strains. Wild-type and *sla1Δ* cells were cultured YPD, loaded with MitoSOX, and then analyzed by flow cytometry. **(F)** Analysis of ROS levels. WT and *sla1Δ* strains were incubated at 30 °C for 12 h, then ROS levels were measured (*n* = 4). **(G)** Analysis of ATP levels. WT and *sla1Δ* strains were incubated at 30 °C, then ATP levels were measured (*n* = 3). **(H)** RT-qPCR analysis of genes involved in mitochondrial respiration and energy metabolism in WT and *sla1Δ* strains (*n* = 3). **(I)** Analysis of ATP and ROS levels. Measurement of relative intracellular ATP and ROS content in laboratory wild-type susceptible strain and five clinical fluconazole-resistant *C. auris* isolates. (*n* = 3). Two-tailed unpaired *t*-tests were used for statistical analyses; data are expressed as the mean ± SD.

To validate redox imbalance and mitochondrial dysfunction in *sla1Δ*, we performed functional assays. Flow cytometry and quantitative fluorescent probe assays showed increased intracellular ROS levels in *sla1Δ* when compared with WT (Figures 3E,F). Consistent with the dysregulated oxidative phosphorylation signature from our transcriptomic data, *sla1Δ* cells exhibited significantly reduced intracellular ATP levels (Figure 3G), and RT-qPCR analysis confirmed downregulation of the key genes involved in mitochondrial respiration and energy metabolism (Figure 3H). We selected five clinical fluconazole-resistant *C. auris* isolates for validation experiments, Consistent with our laboratory generated *sla1Δ* phenotype, these clinical isolates also showed reduced intracellular ATP content and elevated mitochondrial ROS levels relative to the susceptible wild-type strain(Figure 3I).

Collectively, these results establish that *SLA1* deletion disrupts mitochondrial bioenergetics and cellular redox homeostasis, leading to ROS accumulation and ATP depletion. This mitochondrial and redox dysfunction, rather than metal ion dysfunction, acts as an upstream driver of *CDR1*-dependent FLC resistance in *C. auris*.

### *SLA1* deletion drives FLC resistance via mitochondrial ROS-dependent *CDR1* upregulation

The deletion of *SLA1* in *C. auris* induces a unique mitochondrial state, featuring altered redox balance, elevated mitochondrial ROS, and upregulated drug resistance genes (including *CDR1*), ultimately enhancing azole resistance. Notably, this phenotype involves moderate mitochondrial dysfunction rather than complete loss, converting mitochondria into a perturbed but functional state.

Confocal microscopy with Image-iT™ TMRM and Mito-Tracker Green confirmed that *sla1Δ* cells have disrupted mitochondrial networks and mild ΔΨₘ reduction, which is consistent with moderate mitochondrial defect, the TEM observations provide direct ultrastructural evidence for mitochondrial damage in *sla1Δ* cells (Figure 4A). Flow cytometry revealed higher basal ROS in *sla1Δ* than wild-type, with FLC further increasing ROS, peaking in FLC-treated *sla1Δ* cells (Figure 4B).

**Figure 4:**
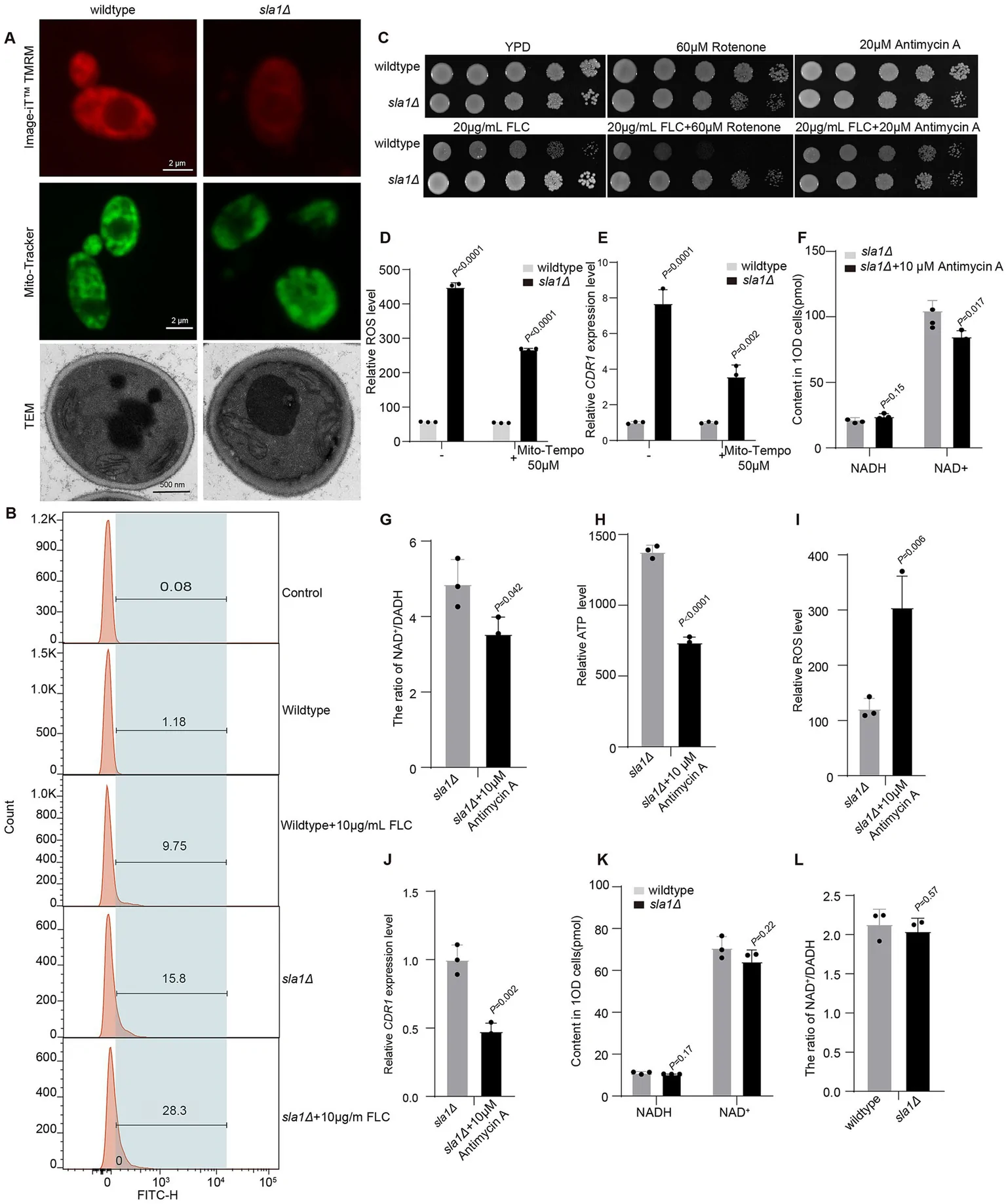
*SLA1* deletion drives FLC resistance via mitochondrial ROS-dependent *CDR1* upregulation in *C. auris*.**(A)** Mitochondrial Membrane Potential, Mito-Tracker and TEM Assay. Representative confocal microscopy and TEM images showing mitochondrial morphology in WT and *sla1Δ*. Scale bars: 2 μm (confocal) and 500 nm (TEM). **(B)** Quantification of cell populations stained with MitoSOX. Wild-type and *sla1Δ* cells were cultured with or without FLC, loaded with MitoSOX, and then analyzed by flow cytometry. **(C)** Spotting assays of WT and *sla1Δ* strains, Strains were spotted onto plates containing YPD, Rotenone, Antimycin A, FLC, FLC + Rotenone, or FLC + Antimycin A. **(D)** Analysis of ROS levels. Relative intracellular ROS levels in WT and *sla1Δ* strains with or without Mito-Tempo treatment. **(E)** Analysis of the mRNA expression levels of *CDR1* by RT-qPCR. mRNA samples (*n* = 3) were isolated from the indicated strains, and cDNAs were synthesized And used for RT-qPCR analysis (*n* = 3) of indicated genes. **(F)** Quantification of intracellular NADH and NAD^+^ levels in *sla1Δ* cells with and without Antimycin A treatment. **(G)** The ratio of NAD^+^/NADH in *sla1Δ* cells with and without Antimycin A treatment. **(H)** Analysis of ATP levels in *sla1Δ* cells with and without Antimycin A treatment (*n* = 3). **(I)** Analysis of ROS levels. *sla1Δ* cells with and without Antimycin A treatment were incubated at 30 °C for 4 h, then ROS levels were measured (*n* = 3). **(J)** Analyses of CDR1 using RT-qPCR. *sla1Δ* strains (*n* = 3) were grown with or without Antimycin A, then RT-qPCR was performed to determine gene expressions of *CDR1* (*n* = 3). **(K)** Quantification of intracellular NADH and NAD^+^ contents in WT and *sla1Δ* strains. **(L)** The ratio of NAD^+^/NADH in WT and *sla1Δ* strains. Two-tailed unpaired *t*-tests were used for statistical analyses; data are expressed as the mean ± SD.

To identify the mitochondrial complex responsible for mtROS overproduction, we inhibited complex I with rotenone and complex III with antimycin A. Spotting assays demonstrated that antimycin A, but not rotenone, recapitulated the FLC resistance of *sla1Δ* cells. Moreover, antimycin A did not further enhance resistance in *sla1Δ*, indicating that *SLA1* deletion and complex III inhibition converge on a shared pathway (Figure 4C). Mitochondria-targeted Mito-Tempo abrogated ROS accumulation and reduced *CDR1* (Figures 4D,E), thereby confirming that mtROS can directly mediate *CDR1* upregulation. Notably, robust antimycin A inhibition of mitochondrial complex III caused profound mitochondrial failure in *sla1Δ* cells, with antimycin A-induced NADH accumulation, ATP collapse (Figures 4F–H). Despite further ROS elevation (Figure 4I), *CDR1* expression and FLC resistance decreased (Figures 4C,J). The basal NADH and NAD^+^ levels in the NAD^+^/NADH ratio did not show significant differences between WT and *sla1Δ* (Figures 4K,L), reflecting perturbed mitochondria’s ability to maintain redox homeostasis. Antimycin A disrupted this balance in *sla1Δ*, reducing the NAD^+^/NADH ratio (Figure 4G), which is consistent with the transition to complete mitochondrial failure.

Collectively, these data support a model wherein *SLA1* deletion converts mitochondria to a perturbed yet functional state and elevated mtROS drives *CDR1* upregulation and FLC resistance. Critically, elevated ROS alone cannot sustain resistance without functional mitochondria, as complete mitochondrial failure abrogates this phenotype despite higher ROS.

### *SLA1* deletion disrupts cell wall and membrane integrity

As previously shown, *SLA1* deletion confers FLC resistance via a mitochondrial ROS–CDR1 efflux axis, and transcriptomic data linked *SLA1* to cell periphery and cytoskeletal organization (Figure 1C). Given that cell surface architecture and efflux pump activity influence antifungal susceptibility, we characterized *SLA1*-dependent alterations in cell wall and membrane integrity to define the resistance mechanism.

Stress sensitivity assays demonstrated that *sla1Δ* exhibited increased susceptibility to Congo Red, sodium dodecyl sulfate, high temperature (39 °C), and hyperosmotic stress, indicating impaired cell wall and membrane integrity (Figure 5A). Transcriptomic profiling and RT-qPCR revealed a significant upregulation of cell wall biogenesis/remodeling genes and core cell wall integrity pathway components in *sla1Δ*, which is consistent with a compensatory stress response (Figures 5B,C).

**Figure 5:**
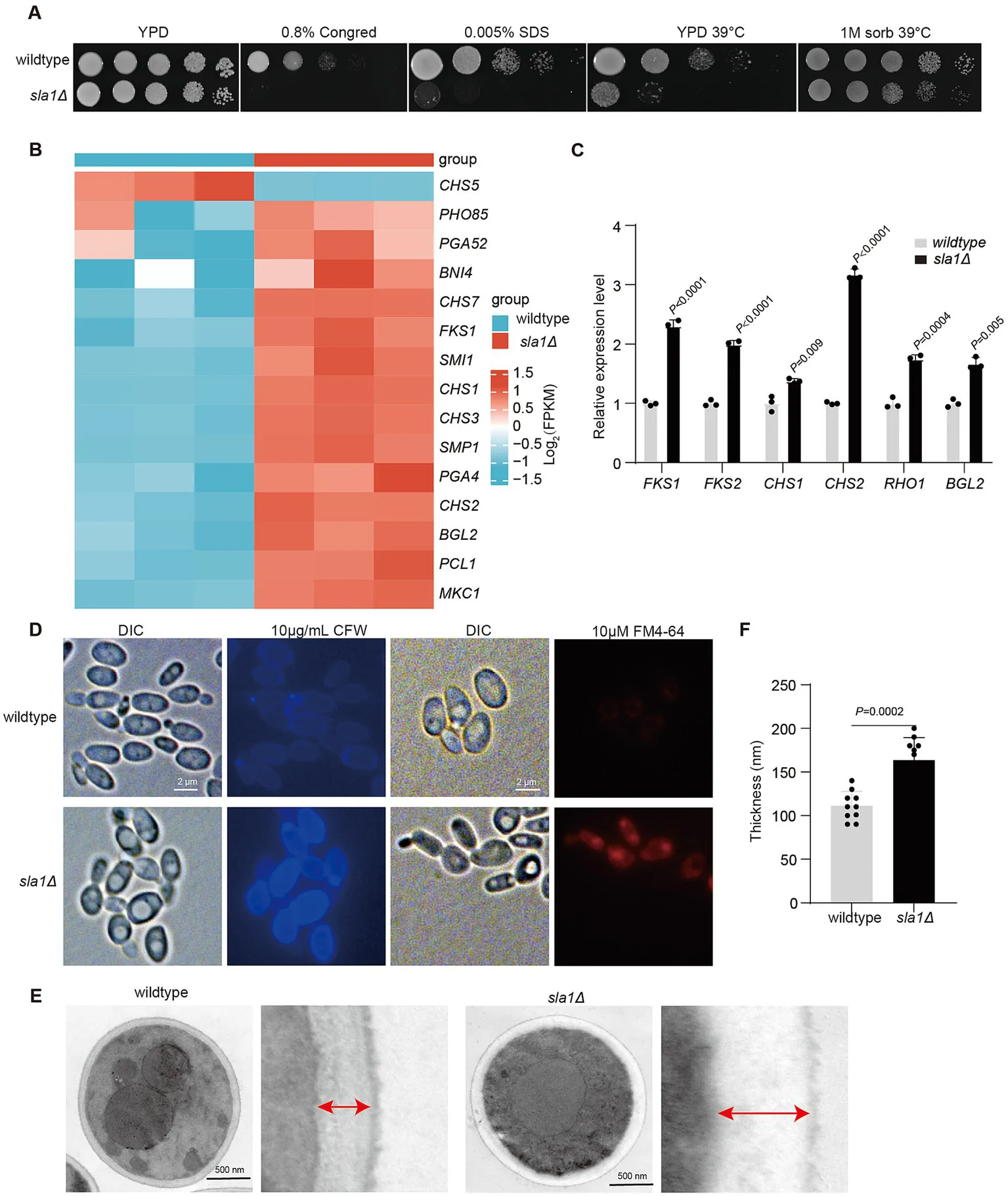
*SLA1* deletion disrupts cell wall and membrane integrity.**(A)** Spotting assays of WT and *sla1Δ* strains. WT and *sla1Δ* strains under cell wall/membrane stress conditions, including Congo Red (Red), SDS, high temperature (39 °C), and high osmolarity (YPD + sorb 39 °C). **(B)** Heatmap of cell wall biogenesis-related genes in WT and *sla1Δ* strains. Samples of RNA were isolated from WT and *sla1Δ* cells (*n* = 3). Transcriptome analysis was performed, and a heat map of the expressions of cell wall biosynthesis genes was generated. **(C)** Analyses of key cell wall biosynthesis genes using RT-qPCR. Indicated strains (*n* = 3) were grown YPD, then RT-qPCR was performed to determine gene expressions of *FKS1*, *FKS2*, *CHS1*, *CHS21*, *RHO1*, and *BGL2.* Two-tailed unpaired t-tests were used. Data are expressed as mean ± SD. **(D)** Microscopic analysis of WT and *sla1Δ* cells using Calcofluor White (CFW) and FM4-64 staining. Scale bars = 2 μm. **(E)** Transmission electron microscopy (TEM) images showing cell wall structure in WT and *sla1Δ* strains. Scale bar = 500 nm. **(F)** The thickness of the cell wall was measured. The cell walls of at least 10 cells were measured in each experiment. Two-tailed unpaired *t*-tests were used for statistical analyses; data are expressed as the mean ± SD.

Fluorescent staining confirmed abnormal chitin distribution (calcofluor white) and disrupted membrane architecture (FM4-64) in *sla1Δ* (Figure 5D). Transmission electron microscopy further revealed an approximately 50% increase in the cell wall thickness in *sla1Δ* when compared to the WT controls (Figures 5E,F).

Together, these data demonstrate that *SLA1* deletion induces compensatory cell wall remodeling, which synergizes with the mitochondrial ROS–*CDR1* efflux axis to amplify FLC resistance. Although these structural changes could alter drug accessibility, the concurrent increase in membrane permeability suggested complex remodeling of the cell envelope. Together with *CDR1*-mediated efflux, these alterations likely contribute to the multifactorial resistance phenotype of *sla1Δ*, although their relative contributions remain to be quantitatively dissected.

### *SLA1* is a key virulence determinant of *Candidozyma auris in vivo*

Our findings established that *SLA1* governs two core cellular processes critical for *C. auris* fitness and drug resistance: mitochondrial homeostasis and cell-wall integrity. To define the impact of these defects on *in vivo* pathogenicity, we assessed *sla1Δ* virulence in a murine model. Fungal burden assays demonstrated significantly reduced colonization of the liver, spleen, and kidney in *sla1Δ*-infected mice at different time points (i.e., 6, 24, 72, and 120 h postinfection), with corresponding reductions in tissue fungal infiltration, as confirmed by histopathology (Figures 6A–C), demonstrating that *SLA1* is essential for *C. auris in vivo* persistence.

**Figure 6:**
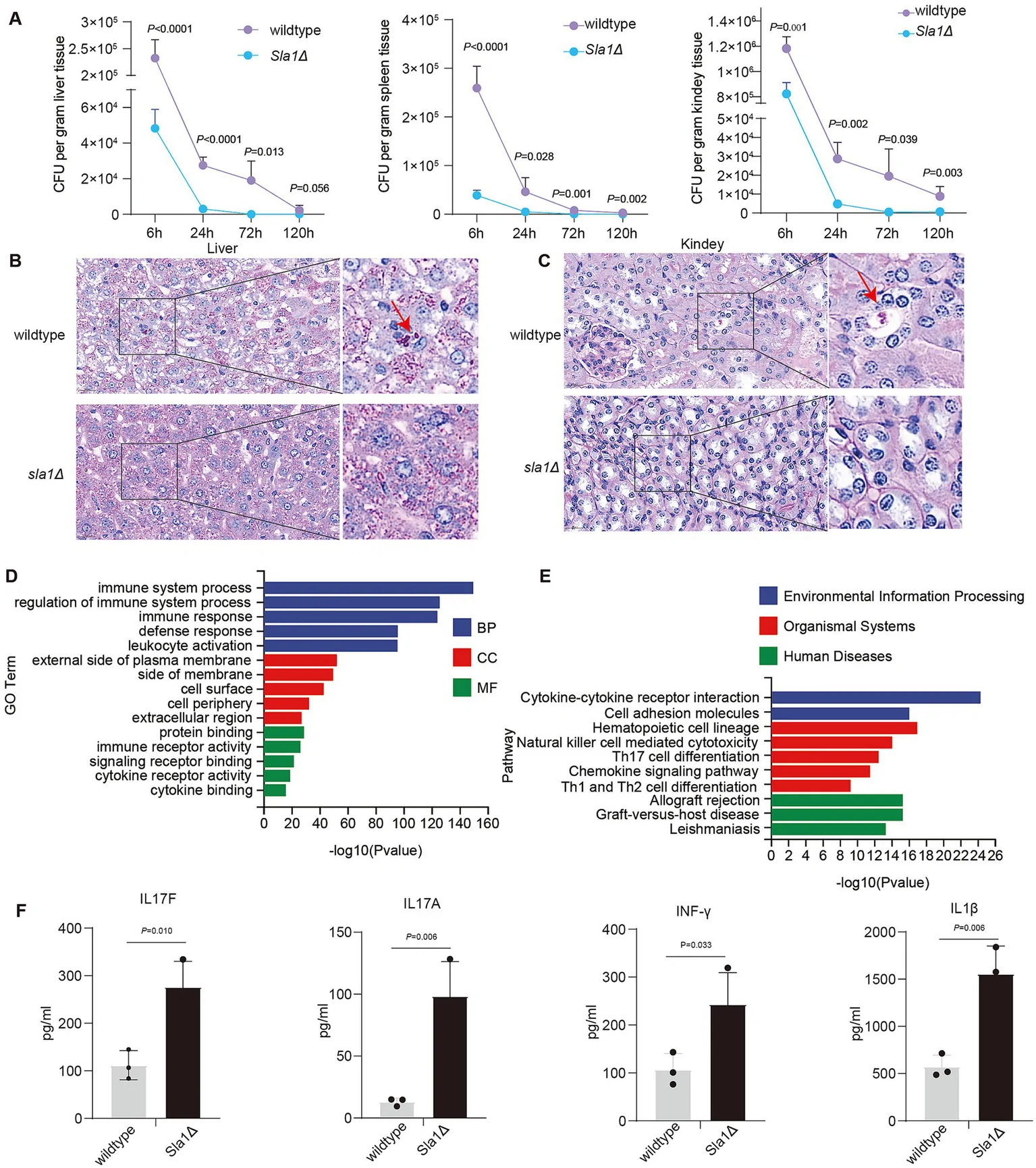
*SLA1* deletion attenuates fungal virulence and enhances host proinflammatory responses. **(A)** Colony-forming unit (CFU) analysis. Mice were infected with indicated fungal strains. CFUs of homogenates of tissues isolated from mice at 6, 24, 72, and 120 h post-infection were enumerated and normalized to tissue weights (*n* > 4). **(B,C)** Histopathological analysis of liver and kidney tissues. Infected liver and kidney tissues were subjected to Periodic Acid-Schiff (PAS) staining and examined using a microscope. Scale bar = 20 μm. Fungal cells are indicated by red arrows. **(D)** Gene GO analyses. GO analyses were performed using RNA-seq data from tissues infected with WT or *sla1Δ* strains. (**E**) Kyoto Encyclopedia of Genes and Genomes (KEGG) analyses. KEGG analyses were performed using RNA-seq data from tissues infected with WT or *sla1Δ* strains. **(F)** ELISA analysis of proinflammatory cytokines (IL-17F, IL-17A, IFN-γ, and IL-1β). Mice infected with wild-type and *sla1Δ* strains were subjected to blood collection for ELISA analysis. Two-tailed unpaired *t*-tests were used for statistical analyses; data are expressed as the mean ± SD.

Host responses to *sla1Δ* infection were further characterized by transcriptomic profiling of infected mouse kidneys. GO and KEGG enrichment analyses revealed the activation of host immune pathways, including leukocyte activation, cytokine–cytokine receptor interaction, chemokine signaling, and Th1/Th2 cell differentiation in *sla1Δ*-infected mice (Figures 6D,E). These findings were validated by significantly elevated serum levels of IL-17F, IL-17A, IFN-γ, and IL-1β in the sla1Δ-infection group (Figure 6F).

Collectively, these data identify *SLA1* as a key virulence determinant in *C. auris.* The attenuated *in vivo* pathogenicity of *sla1Δ* reflects a combined mitochondrial dysfunction and cell wall defects that impair fungal fitness and enhance host immune recognition. This dual phenotype (impaired fungal persistence with heightened host proinflammatory immunity) highlights *SLA1* as a promising therapeutic target to address azole resistance while boosting antifungal host defense.

## Discussion

Invasive infections caused by *C. auris* have become a major global health concern, driven by rapid acquisition of multidrug resistance and widespread resistance to FLC (Lass-Flörl et al., 2024). Although canonical mechanisms such as mutations in *ERG11* and the overexpression of efflux pumps, including *CDR1,* are well-established, these genetic determinants alone do not fully account for the high prevalence and plasticity of azole resistance observed in clinical isolates (Barker et al., 2026; Carolus et al., 2024; Hong et al., 2023). Increasing evidence suggests that host-derived stresses, particularly oxidative stress, play a critical role in shaping antifungal resistance (Areitio et al., 2026; Kamli et al., 2022; Rana and Thakur, 2025); however, how such environmental cues are integrated into resistance programs in *C. auris* remains incompletely unclear (Song Y. et al., 2026).

In this study, we identified *SLA1* as a previously unrecognized regulator of oxidative stress–mediated antifungal resistance through transcriptomic analysis. We further delineated a regulatory axis in which *SLA1* deletion induces mitochondrial complex III dysfunction, promoting increased mitochondrial ROS production and subsequent upregulation of resistance-associated gene transcription, Intriguingly, excessive disruption of mitochondrial activity led to elevated ROS levels; however, it markedly reduced *CDR1* transcription and fully abrogated azole resistance, concurrent with NADH buildup, ATP exhaustion and impaired respiratory proliferation (Figure 7). In addition, *SLA1* was demonstrated to contribute to cell wall homeostasis and regulate virulence in *C. auris*. These findings provide an insight into the molecular basis of multidrug resistance and identify potential therapeutic targets for combating *C. auris* infections. Furthermore, the *sla1Δ* strain exhibited a distinct antifungal susceptibility profile, with strong resistance to azoles and high sensitivity to other first-line antifungal agents, including amphotericin B and echinocandins. This phenotype may have implications for therapeutic strategies in managing drug-resistant *C. auris* infections.

**Figure 7:**
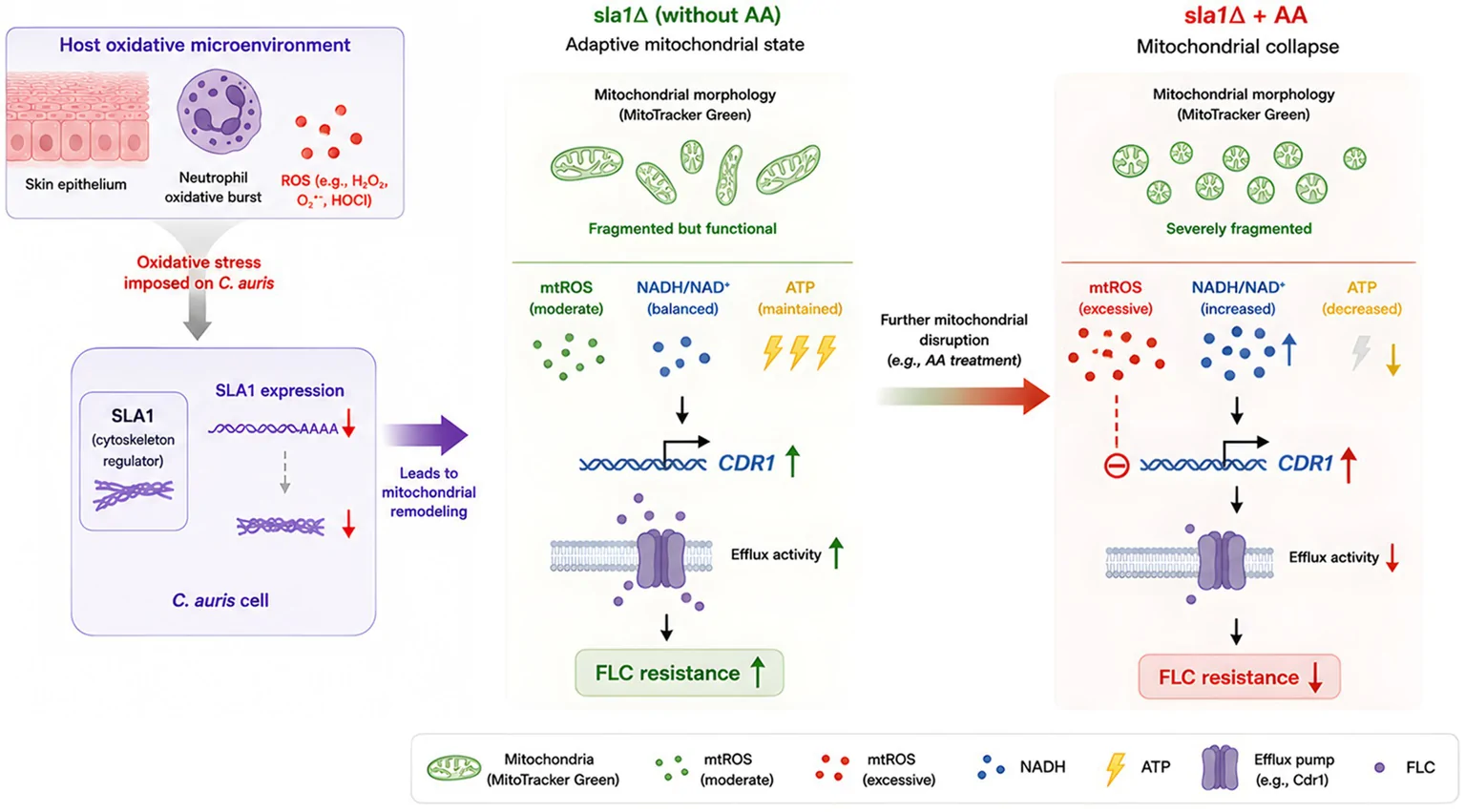
Model diagram of the *SLA1* mediating FLC resistance in *C. auris*. High-oxidative stress from the external environment significantly downregulates *SLA1* expression in *C. auris*. *SLA1* deletion induces a “partially damaged yet functionally competent” mitochondrial state, which drives the upregulation of *CDR1* via mtROS signaling, enhances efflux pump activity, and consequently promotes FLC resistance. In contrast, antimycin A (AA)-induced mitochondrial collapse leads to excessive mtROS accumulation, which paradoxically inhibits *CDR1* expression and reverses FLC resistance. These findings demonstrate that mtROS-mediated drug resistance signaling is strictly dependent on mitochondrial functional integrity.

A key insight from our work is that mitochondrial ROS-driven transcriptional activation of resistance genes can be mechanistically uncoupled from the execution of antifungal resistance. Under *SLA1*-deficient conditions, elevated mtROS is sufficient to induce *CDR1* expression and promote drug resistance. This state-dependent concept is further corroborated by clinical fluconazole-resistant *C. auris* isolates, which recapitulate the *sla1Δ* signature: lowered ATP and enhanced mtROS. However, this ROS-centric model is challenged by our observations under conditions of severe mitochondrial perturbation. Pharmacological inhibition of mitochondrial complex III caused a marked accumulation of NADH and collapse of ATP production, collectively indicating functional failure of the mitochondria. Strikingly, despite a further increase in ROS levels under these conditions, *CDR1* expression and FLC resistance were significantly reduced. These findings demonstrated that ROS elevation alone is insufficient to sustain antifungal resistance in the absence of functional mitochondrial capacity.

Together, these results support a model wherein the biological outcome of mitochondrial ROS is determined by the functional state of the mitochondria. In cells with partially impaired but operational mitochondria, mtROS acts as a signaling intermediate that promotes resistance gene expression. In contrast, when mitochondrial function is severely compromised, ROS no longer serves as an effective signaling molecule, but rather reflects dysfunctional electron transport and metabolic collapse. Under these conditions, the cellular systems required to execute resistance, including energy supply, membrane trafficking, and efflux activity, are impaired, leading to the loss of resistance despite elevated oxidative stress. This state-dependent model revises the prevailing view of ROS as a uniformly pro-resistance factor and instead positions mitochondrial integrity as the critical determinant of whether ROS can function as a regulatory signal.

An important unresolved question is how *SLA1*, a protein primarily implicated in actin organization and endocytic trafficking, influences mitochondrial function. Our data favor an indirect, system-level mechanism in which *SLA1* maintains the cellular architecture required for organelle homeostasis. The disruption of *SLA1* likely perturbs cytoskeleton-dependent processes such as organelle positioning, membrane contact site organization, or vesicular transport, which, in turn, compromise mitochondrial integrity (Liu et al., 2026; Wu and Chan, 2024). In this framework, mitochondrial dysfunction in *sla1Δ* cells is not the result of a linear signaling pathway but rather emerges from global defects in intracellular organization, highlighting a functional link between cytoskeletal dynamics and mitochondrial homeostasis.

At the transcriptional level, our findings further suggest the existence of a noncanonical, redox-sensitive regulatory layer controlling *CDR1* expression. Specifically, the loss of *SLA1* causes selective impairment of the mitochondrial complex III, leading to electron leakage and elevated mtROS production, which, in turn, induces transcriptional upregulation of *CDR1*. This effect is independent of canonical *CDR1* regulators such as *CAP1*, *TAC1*, and *GSK3*, as well as major stress-responsive transcription factors (i.e., *HOG1*, *SKN7*, *MSN2*), as indicated by our transcriptomic data (Czajka et al., 2023; Barker et al., 2025b; Jacobs et al., 2022; Peng et al., 2024; Ranjbar Golafshani et al., 2025). Notably, analysis of clinical *C. auris* isolates revealed a significant negative correlation between the expression levels of *SLA1* and *CDR1*, further supporting the clinical relevance of this regulatory pattern. The precise molecular mechanism linking mtROS to *CDR1* transcriptional activation remains undefined. We accordingly hypothesized that mtROS may activate *CDR1* through (i) an-unidentified redox-sensitive transcription factor, (ii) post-translational modification of the existing transcriptional machinery, or (iii) epigenetic remodeling of the *CDR1* locus. These possibilities warrant future investigation using chromatin accessibility profiling and proteomic approaches. Importantly, our data indicate that such redox-dependent regulation operates only within a permissive mitochondrial context, reinforcing the notion that signaling and metabolic capacity are tightly coupled; that is, mitochondrial redox state may influence gene expression through alternative mechanisms only when the mitochondrial function is sufficiently preserved (Liu et al., 2025; Perez-Castillo et al., 2021; Yang et al., 2026).

Beyond antifungal resistance, *SLA1* deletion also affects cellular integrity and pathogenic fitness. Increased membrane permeability and reduced virulence in *sla1Δ* cells suggest that the adaptive advantages conferred by elevated ROS and resistance gene activation are accompanied by physiological costs. This trade-off is consistent with the broader principles of microbial adaptation, in which resistance to one form of stress may compromise fitness under other conditions. In the case of *sla1Δ*, mitochondrial perturbation appears to enhance survival under antifungal pressure while simultaneously impairing cellular robustness and host-interaction capacity.

Several limitations of this study warrant consideration. First, the molecular mechanisms linking *SLA1* to mitochondrial homeostasis remain to be elucidated and warrant further investigation into cytoskeleton–organelle interactions. Second, the identity of the redox-sensitive factors mediating mtROS-dependent transcriptional responses remains unknown. Third, although we observed a significant negative correlation between *SLA1* and *CDR1* expression in clinical isolates, the present study lacks paired fluconazole MIC phenotypic data for these clinical strains. Further validation with combined transcriptional and drug susceptibility profiling is required to fully consolidate the clinical relevance of this regulatory axis. Although our data consistently associate *SLA1* deletion with mitochondrial dysfunction, ROS accumulation and elevated *CDR1* expression, the precise molecular events linking these processes remain to be determined. Given the established role of *SLA1* in membrane trafficking and cytoskeletal organization, it is possible that additional mechanisms contribute to the observed phenotype. Future studies will be required to define the upstream regulators responsible for *CDR1* activation. Finally, considering that *C. auris* infections predominantly occur in immunocompromised hosts, future studies in immunodeficient models will be important to fully assess the *in vivo* implications of *SLA1*-mediated regulation.

In summary, this study defines a mitochondrial state-dependent framework for antifungal resistance in *C. auris*. We demonstrated that mitochondrial ROS promotes resistance gene induction only when mitochondrial function is preserved, whereas the loss of mitochondrial integrity abolished resistance despite elevated oxidative stress. This uncoupling of resistance induction and execution expands the current models of antifungal resistance and identifies mitochondrial homeostasis as a critical determinant, and potential therapeutic vulnerability, in multidrug-resistant *C. auris*.

## Materials and methods

### Ethical statements

All animal experiments were reviewed and ethically approved by Laboratory Animal Welfare and Ethics Committee of the Army Medical University (AMUWEC20265557). The Chinese Ministry of Science and Technology’s Guide for the Care and Use of Laboratory Animals governed all animal studies. Female C57BL/6 mice aged 6–8 weeks were obtained from Chongqing Laibite Biological Technology Co., Ltd. All mice had access to unlimited food and water in a quiet, specific-pathogen-free environment.

### Strains and growth conditions

All strains are listed in Table 1. Strains were grown in YPD (1% yeast extract, 2% peptone, 2% glucose), The transformant selection was performed using YPD agar supplemented with 200 μg/mL nourseothricin(Jena Bioscience, cat# AB-101) and incubated at 30 °C or 1,000 μg/mL G418 (Thermo Fisher, cat# 11811031). Drug-resistance tests were performed using 20 μg/mL FLC (MedChemExpress, cat# HY-B0101), 0.2 μg/mL ketoconazole (KTC) (MedChemExpress, cat# HY-B0105), 1.0 μg/mL Amphotericin B (AMB) (MedChemExpress, cat# HY-B0221), 10 mM NAC (Sigma, cat# 1009005), 20 μM Antimycin A (Sigma, cat# 77332), 50 μM Mito-TEMPO (MedChemExpress, cat# HY-112879), 60 μM Rotenone (MedChemExpress, cat# HY-B1756), and 1 mM H₂O₂ (Sigma, cat# 108600).

**Table 1:** *Candida auris* strains and plasmids used in this study.

(A) *Candidozyma auris* strains used in this study			
Lab ID	Background strains	Genotype	Reference
SC99	*Candida auris* 2,852		Gift from Chen Ding
SC100	*Candida auris* 2,852	*sla1∆*	This Study
SC101	*Candida auris* 2,852	*cdr1∆*	This Study
SC102	*sla1∆*	*sla1∆cdr1∆*	This Study
CDCL201	*Candida auris*		Gift from Chen Ding
CDCL202	*Candida auris*		Gift from Chen Ding
CDCL203	*Candida auris*		Gift from Chen Ding
CDCL204	*Candida auris*		Gift from Chen Ding
CDCL205	*Candida auris*		Gift from Chen Ding
CDCL206	*Candida auris*		Gift from Chen Ding
CDCL207	*Candida auris*		Gift from Chen Ding
CDCL208	*Candida auris*		Gift from Chen Ding
CDCL209	*Candida auris*		Gift from Chen Ding
CDCL210	*Candida auris*		Gift from Chen Ding
CDCL211	*Candida auris*		Gift from Chen Ding
CDCL212	*Candida auris*		Gift from Chen Ding
CDCL213	*Candida auris*		Gift from Chen Ding
CDCL214	*Candida auris*		Gift from Chen Ding
CDCL215	*Candida auris*		Gift from Chen Ding
CDCL216	*Candida auris*		Gift from Chen Ding

(B) Plasmids used in this study		
Plasmid	Description	Source
pTO135	pENO1-CaCas9-tCyc1, ampR	Purchase from Addgene
pTO128	CaNAT1R in T-DNA region, kanR	Purchase from Addgene
pTO149	RFP-tADH1-pAgTEF2-NEO-tAGTEF2, with targeting arms to tag Eno1 cterminus, natR ampR	Purchase from Addgene
pTO136	pADH1-tRNA-Ala-gRNA-tracrRNA-HDV-tAgTEF2, ampR	Purchase from Addgene

### Generation of fungal mutants

All primers are listed in Table 2. The *sla1∆* strains were generated by the CRISPR-Cas9 system (Santana and O'Meara, 2021; Schulze et al., 2026). A linear Cas9 cassette was amplified from pTO135 using primers 1–2, linear repair cassettes were amplified as follows: Primers 7 and 8 were used to amplify the upstream sequence, Primers 11 and 12 were used to amplify the downstream sequence, and primers 9 and 10 were used to amplify the selectable marker (*NAT*) from pTO128, then primers 7 and 10 were used to link the three PCR fragments. sgRNA were amplified as follows: Fragment 1 was amplified from pTO136 using primers 3–6, Fragment 2 was amplified from pTO136 using primers 4-5, then primers 3 and 4 were used to link the two PCR fragments. All linear PCR products were purified with the Zymo DNA Clean & Concentrator kit (Zymo Research, cat no. D4034,) following the manufacturer’s instructions. The resulting PCR fragments were purified and transformed into the WT strains using electroporation. Transformants were selected on YPD + NAT medium. Correct integration was verified using PCR and real-time PCR. The same method was used to construct the *CDR1∆* strain.

**Table 2:** Oligonucleotide primers used in this study.

Number	Name	Sequence (5′ to 3′)
1	CaCAS9 F	CCTCTTTGTAGTTCAACTTATGC
2	CaCAS9 R	GTCCCAAAACCTTCTCAAGC
3	sgRNA Fusion F1	GCTATTACGCCAGCTGG
4	sgRNA Fusion R2	CGCAATTAATGTGAGTTAGC
5	Fusion PCR-SLA1 gRNA	CTTGTACGACTACCAAGCCCgttttagagctagaaatagcaag
6	Fusion PCR-SLA1 gRNA	GGGCTTGGTAGTCGTACAAGTGGACGAGTCCGGATTC
7	SLA1 UP F	ATGTGTTTGAGAAAGAGCCTGCT
8	SLA1 UP R	GATGGAGCAGGGCAAAGAAG
9	NAT F	TAGAGCTTCTTTGCCCTGCTCCATCCGTCAAAACTAGAGAATAATAAAGAAAAC
10	NAT R	gaccacctttgattgtaaatagt
11	SLA1 Down F	actatttacaatcaaaggtggtcCCATTTGGCTTCTGAATGACC
12	SLA1 Down R	CGTTGATGATCTTCCTCACCTT
13	SLA1 CHECK F	AACTCCATGTCGAAGCCTTTTAG
14	Fusion PCR-CDR1 gRNA F	CAAGTGCTGCCCCATGACAAgttttagagctagaaatagcaag
15	Fusion PCR-CDR1 gRNA R	TTGTCATGGGGCAGCACTTGTGGACGAGTCCGGATTC
16	CDR1 UP F	ATGATAACCCTCTAGGCACCG
17	CDR1 UP R	GGAGATGGAAAAGTGAAGATTT
18	NEO F	AAATCTTCACTTTTCCATCTCCCGACATGGAGGCCCAGAATA
19	NEO R	CAGTATAGCGACCAGCATTCACA
20	CDR1 Down F	atgtgaatgctggtcgctatactgAGTGCATACATTAATACGATAAGTCGT
21	CDR1 Down R	AAATATCACTTAACGCAAAAGGAC
22	CDR1 CHECK	ACTCAATGCTCGTTTAGAATCCAA
23	B9J08_00927 qPCR F	AGGGCTTCGTTGCTCTCTTA
24	B9J08_00927 qPCR R	GCTGGTAGCCCAAACCTGTA
25	B9J08_01160 qPCR F	TCAGGAGGTAAATCCAGCGA
26	B9J08_01160 qPCR R	CAAGTCAAAGAAGAAAGCTCTTGC
27	B9J08_02104 qPCR F	AGCCTCCCCAAAGCTTCAAT
28	B9J08_02104 qPCR R	GCAGCTTGCAAATTGGCCC
29	B9J08_03279 qPCR F	CGCAGCAGAGCATTGATGAT
30	B9J08_03279 qPCR R	GTATATCCACCGGCACCAGG
31	B9J08_04096 qPCR F	TCAACAGTACCTCACCATCACA
32	B9J08_04096 qPCR R	TCACCAATCGTCATCATCTTCGT
33	B9J08_05040 qPCR F	TCCACCTGTTCCAGAAGTGC
34	B9J08_05040 qPCR R	GAGAAGGGGCAGCCTTTGAT
35	ACT qPCR F	ACGCACATCGACATCACATT
36	ACT qPCR R	CCTCTCAGTCGTCCGCTATC
37	ERG11 qPCR F	TGATGCCTCCTTCGCCAAAT
38	ERG11 qPCR R	AGCTGAAGTGTGCTGACCTC
39	ERG3 qPCR F	TTGGCAGCAAAAACGCTACC
40	ERG3 qPCR R	GCAGTAGTAGCACGCCAGAT
41	ERG6 qPCR F	GCCTACGGTATCGAGGTTGG
42	ERG6 qPCR R	CTCCATCAACCCGACAAGCT
43	ERG2 qPCR F	GACTGGGTGTTCAACAACGC
44	ERG2 qPCR R	ACTGCTTCACTTGGCCTCTC
45	CDR1 qPCR F	TGGTGACTGCGTTCTACGTC
46	CDR1 qPCR R	TGGCCACAAGTTTGACCACT
47	*PMC1* qPCR F	CTGTTGCCTCTTCAAACGGC
48	*PMC1 qPCR R*	AAAGGCATCGTCCTACCTGC
49	GSK3 qPCR F	TGGCAGCAAGGTAACCACAG
50	GSK3 qPCR R	CGGTTCTTAAATCGCTTGTCCTG
51	CAP1 qPCR F	CTACCATCGGACGTCAGCTC
52	CAP1 qPCR R	CGTCCTTCTGGTCCAACACA
53	TAC1 qPCR F	AAGCGCTAACCTCAGCAACT
54	TAC1 qPCR R	GATCGAAATGCCCGTGCTTC
55	CHS1 qPCR F	TCTATCAGGGCGAGGAGGTC
56	CHS1 qPCR R	TGCGCTTGTGTGACCATTTG
57	CHS2 qPCR F	CTGGCACATGTTCACCTCCT
58	CHS2 qPCR R	CCTCCATCGTCGTCTCTTGG
59	BGL2 qPCR F	TTCTCGTAGATGGCGGTTCG
60	BGL2 qPCR R	TTCTTGGCATTCTCCACGCT
61	FKS1 qPCR F	AGTTCGCCGGTGACTTCATT
62	FKS1 qPCR R	TCGGCGATGAATCTACCAGC
63	FKS2 qPCR F	CAGTGCTCCTTGGCCCAATA
64	FKS2 qPCR R	GCAAACGAACTCACGGATCG
65	RHO1 qPCR F	CAGATTGAGGCCCTTGTCGT
66	RHO1 qPCR R	GGATGCTCTTGTGGCAGTCT
67	B9J08_00689 qPCR F	GCGATCGTTACGATGTCCCT
68	B9J08_00689 qPCR R	CGCACCAATTTTGCAATCGC
69	B9J08_01470 qPCR F	GACCATCTTGGGCCTTTCGA
70	B9J08_01470 qPCR R	GAAGACCTTCGTCCTTGGCA
71	B9J08_02659 qPCR F	GGTGCATGGGACCTTACGAA
72	B9J08_02659 qPCR R	ACGCACTTATCTGGTCCACG
73	B9J08_02698 qPCR F	TGCCACACAGAACGAGGTTT
74	B9J08_02698 qPCR R	GGGCCATCTCCAAACCAGAA
75	B9J08_05509 qPCR F	CTGGAGACCTCGCGAAGTAC
76	B9J08_05509 qPCR R	GCCCACCCTGCTGACTTTAT
77	B9J08_01909 qPCR F	ACAGTTGGATGGATCTGGCC
78	B9J08_01909 qPCR R	CACTGGCCCAAAGTTTCACG
79	B9J08_00130 qPCR F	ACCAGAGCCAAGTTCGCTAC
80	B9J08_00130 qPCR R	AGTTGTTGTTGGCCTGTGGA
81	B9J08_05430 qPCR F	TGGAGCAGCCAGTGTACTTG
82	B9J08_05430 qPCR R	CACCCAAAGCCTTGAACACG
83	B9J08_05308 qPCR F	GTGAACGGCAACTTGGGTTC
84	B9J08_05308 qPCR R	AACCTCCTTCTTGATGGCGG
85	B9J08_00211 qPCR F	GTGGTGGTAGCGGATACTCG
86	B9J08_00211 qPCR R	ACATCCTTTTCGAGCACCGT
87	B9J08_00750 qPCR F	CGCGTATACCAACGTGCAAG
88	B9J08_00750 qPCR R	GCACTAATCCGAGGGATCCG
89	B9J08_02404 qPCR F	AGGACTTGCACTCGACCAAG
90	B9J08_02404 qPCR R	AGAGAGCTCCTCACGCCTAA
91	B9J08_00488 qPCR F	AGCAGGTGGGGGTCAGATAT
92	B9J08_00488 qPCR R	CGCAGGTGTAAGTGGACAGT
93	B9J08_01969 qPCR F	GGAGGTGCCCGGTATTTTCA
94	B9J08_01969 qPCR R	GTTGTTGACGAGGTAGGCCA

### Transformation of *Candidozyma auris* by electroporation

Transformation of *Candidozyma auris* via electroporation was performed as described previously (Zhao et al., 2025). Briefly, *C. auris* was grown aerobically in 50 mL of YPD medium at 30 °C with shaking. to exponential phase. Cells were harvested by centrifugation and resuspended in TE buffer with 100 mM lithium acetate then incubated with constant shaking at 30 °C for 1 h. DTT was added to the cells at a final concentration of 25 mM and incubation was continued for 30 min at 30 °C with constant shaking. The cells were harvested by centrifugation; washed once with ice-cold, sterile, ultrapure water; washed once with ice-cold 1 M sorbitol; then resuspended in ice-cold 1 M sorbitol. To perform electroporation, 45 μL of competent cells were mixed with 500–1,000 ng of each PCR-amplified cassette, Cas9, sgRNA, and the repair template, and then added to a pre-chilled 2 mm gap electroporation cuvette. Cells were then electroporated with a Bio-Rad MicroPulser Electroporator. After electroporation, cells were recovered in 1 M sorbitol, resuspended in 10 mL YPD, and allowed to outgrow at 30 °C with continuous rotation for 2 h. The outgrown cells were spread on selective media.

### RT-qPCR

Wild-type and mutant strains were cultured overnight at 30 °C. The cultures were then diluted to an OD600 of 0.2 in fresh YPD and incubated for 4 h. Total RNA was isolated from cell lysates prepared via bead beating using the E.Z.N.A. Total RNA Kit I (Omega Bio-tek, cat# R6834). First-strand cDNA was synthesized using the HiScript III RT SuperMix for qPCR (Vazyme, cat # R323-01). Quantitative real-time PCR was performed with ChamQ SYBR qPCR Master Mix (Vazyme, cat # Q311-02) on a CFX Connect real-time PCR detection system (Bio-Rad).

### Animal infection

Mice were housed at 22 °C with a 12 h light–dark cycle. For intravenous infections, mice were anesthetized with a single intraperitoneal injection of 2.5% 2,2,2-tribromoethanol (meilunbio, cat # MB2548) at a dose of 250 mg/kg body weight, and *C. auris* cells (10^7^ cells in 100 μL PBS) were injected intravenously. Livers, spleens, and kidneys were harvested at 6, 24, 72, and 120 h post-infection from mice that were euthanized by CO₂ exposure. All surviving mice at the experimental endpoint underwent CO₂ inhalation euthanasia following the American Veterinary Medical Association (AVMA) humane animal care guidelines: gradual CO₂ displacement at a flow rate of 1.5 L/min for a minimum of 10 min, followed by secondary cervical dislocation to confirm complete euthanasia. All animal operations were performed under isothermal sterile conditions to minimize animal suffering. and tissue homogenates were obtained. Following serial dilution, the homogenates were plated onto YPD agar, and fungal colony-forming units (CFUs) were counted after incubation at 30 °C for 2 days.

### Enzyme-linked immunosorbent assay (ELISA)

Blood was collected from mice infected with wild-type and mutant strains, from which the serum concentrations of target cytokines/proteins were quantified using commercial ELISA kits (Absin Bioscience Inc., Shanghai, China) as per the manufacturer’s instructions. Briefly, all reagents and serum samples were equilibrated to room temperature before use. The diluted standards and samples were added to the plate in duplicate and incubated at 37 °C for 2 h, followed by sequential incubation with biotinylated detection antibody and HRP-conjugated avidin, with repeated washing steps between the incubations. Color development was examined using TMB substrate in the dark at 37 °C, and the reaction was terminated with sulfuric acid. The absorbance was measured at 450 nm by using a microplate reader.

### Transmission electron microscopy (TEM)

Cells were grown overnight in YPD medium at 30 °C. Wild-type and mutant cells were harvested after a 4-h growth period in liquid YPD medium and then subjected to fixation using 3% paraformaldehyde. The fixed samples were treated with 1% osmium tetroxide. After fixation, the cells were dehydrated through an ethanol gradient and infiltrated with Embed 812 resin. Ultrathin sections were contrasted with uranyl acetate and lead citrate, and then visualized using electron microscope. For each strain, images were captured from multiple cells.

### ROS quantification

*Candidozyma auris* cells were cultured overnight in YPD medium at 30 °C with shaking, the culture was adjusted to an OD₆₀₀ of 0.2 and induced for another 4 h. DCFH-DA probe (Beyotime, cat # S0034S) was diluted 1:1000 with probe dilution buffer to a final concentration of 10 μmol/L. The induced cells were collected and resuspended in the diluted DCFH-DA solution, with the cell concentration adjusted to 1 × 10^6^–2 × 10^7^ cells/mL. The cell suspension was incubated in a 37 °C cell incubator for 20 min, and gently inverted and mixed every 3–5 min during incubation to ensure full contact between the probe and the cells. After incubation, the fluorescence intensity of the cells before and after stimulation was detected in real time or at successive time points using an excitation wavelength of 488 nm and an emission wavelength of 525 nm.

### NAD+/NADH quantification

The WT and mutant strains were inoculated into 10 mL YPD medium and incubated overnight at 30 °C. The samples were diluted to an OD_600_ of 0.2 with YPD medium and then incubated at 30 °C for 4 h. The cells were lysed using a bead beater and then centrifuged to collect the supernatant as the sample to be tested. Subsequently, the sample, WST-8 reaction solution (Beyotime, cat # S0175), and enzyme mixture were added into a 96-well plate in proportions specified in the instruction manual, followed by thorough mixing. The reaction plate was incubated at 37 °C in the dark for 30–60 min, and the absorbance at 450 nm was measured by using a microplate reader. Finally, a standard curve was plotted based on the absorbance of the standards to calculate the NAD^+^/NADH concentrations in the samples, and the data were normalized using the bacterial CFU count or sample protein concentration.

### ATP quantification

For the measurement of the ATP level, the cells were prepared as described earlier. The strains were incubated at the indicated temperatures for 30 min and then disrupted using glass beads. The ATP levels were quantified using the ATP Assay Kit (Beyotime, cat # S0026B) in accordance with the manufacturer’s instructions. The luminescent signals were detected using a Synergy H4 microplate reader (BioTek). All ATP data were normalized to the OD_600_ of each sample, determined via a colorimetric assay and measured using a MultiskanGO microplate reader.

### Staining

To visualize the mitochondria, wild-type and *sla1Δ* strains were cultured overnight and then subcultured to an OD_600_ of 1. The cells were washed twice with PBS and then transferred into preheated Eppendorf tubes containing fresh YPD medium and a fluorescent dye (Image-iT™ TMRM; thermo) or (Mito-Tracker; Beyotime). The strains were washed three times with PBS, and fluorescence images were taken using a Leica stellaris5.

### ROS detection by flow cytometry

To measure the intracellular and mitochondrial ROS levels, wild-type and *sla1Δ* strains were cultured overnight in YPD medium to an OD₆₀₀ of 1. The intracellular ROS level was detected by using a fluorescent probe DCFH-DA (Beyotime), whereas the mitochondrial ROS level was evaluated using MitoSOX™ Red (Invitrogen,cat # M36008). The cells were washed twice with PBS, resuspended in YPD containing the corresponding probe, and incubated at 30 °C for 30 min according to the manufacturer’s protocols. After incubation, the cells were pelleted, washed thrice with PBS, and resuspended in PBS for flow cytometry. The stained and unstained control groups were included in all experiments. Data were acquired using a BD LSRFortessa cell analyzer and analyzed using FlowJo software.

### Data availability

All RNA sequencing data in this study are available at the Gene Expression Omnibus with accession numbers PRJNA1458866, PRJNA1458307 and PRJNA1458183. Any other data necessary to support the conclusions of this study are available from the authors upon request.

### Quantification and statistical analyses

All statistical analyses were performed using the GraphPad Prism software (GraphPad Software Inc., San Diego, CA, USA). All experiments were performed with at least three independent biological replicates. Data are presented as mean ± standard deviation (SD). Statistically significant differences between the two groups were determined using an unpaired two-tailed Student’s *t-*test. Multi-group comparisons using One-way ANOVA. Significant differences were defined as *p* < 0.05, *p*-values rounded to exactly three decimal places. All experiments were performed using three biological replicates to ensure the reproducibility.

## Data Availability

The datasets presented in this study can be found in online repositories. The names of the repository/repositories and accession number(s) can be found in the article/supplementary material.
